# Data on association of mitochondrial heteroplasmy with carotid intima-media thickness in subjects from Russian and Kazakh populations

**DOI:** 10.1016/j.dib.2020.105136

**Published:** 2020-01-14

**Authors:** Tatiana V. Kirichenko, Yulia I. Ragino, Mikhail I. Voevoda, Saule J. Urazalina, Zukhra B. Khasanova, Varvara A. Orekhova, Vasily V. Sinyov, Margarita A. Sazonova, Alexander N. Orekhov, Igor A. Sobenin

**Affiliations:** a Research Institute of Human Morphology, Moscow, Russia; b National Medical Research Center of Cardiology, Moscow, Russia; c Institute for Atherosclerosis Research, Skolkovo Innovative Center, Moscow, Russia; d Research Institute of Internal and Preventive Medicine – Branch of the Institute of Cytology and Genetics, Siberian Branch of Russian Academy of Sciences, Novosibirsk, Russia; e Federal Research Center of Fundamental and Translational Medicine, Novosibirsk, Russia; f JSK Scientific-Research Institute of Cardiology and Internal Diseases, Almaty, Kazakhstan; g Institute of General Pathology and Pathophysiology, Moscow, Russia

**Keywords:** Mitochondrial mutations, Heteroplasmy, Carotid atherosclerosis, Intima-media thickness, Cardiovascular risk factors

## Abstract

The search for variants of mitochondrial genome associated with atherosclerosis, in particular, with carotid intima-media thickness (cIMT), is necessary to understand the role of the damage of mitochondrial genome in the development of atherosclerosis. Such data can be useful to provide novel genetic markers of predisposition to atherosclerosis and molecular targets for further development of technologies aimed to prevent age-related degenerative pathologies. Data presented in this article demonstrate the association of several heteroplasmic variants of mitochondrial DNA (mtDNA) previously described as proatherogenic ones with cIMT in 251 participants (190 participants from Novosibirsk, Russia, and 61 participant from Almaty, Kazakhstan). It was shown that the occurrence of some variants of mitochondrial genome is different in samples derived from Russian and Kazakh populations; the level of mitochondrial heteroplasmy m.13513G > A correlates negatively with mean cIMT in both Russian and Kazakh participants.

## List of abbreviations

CVD: cardiovascular disease
PCR: polymerase chain reaction
cIMT: carotid intima-media thickness
BMI: body mass index
SBP: systolic blood pressure
DBP: diastolic blood pressure
HDL: high-density lipoproteins
LDL: low-density lipoproteins
TG: triglycerides

**Table:** Specifications Table

Subject	Cardiovascular diseases
Specific subject area	Genetic predisposition to carotid atherosclerosis
Type of data	Tables and figures
How data was acquired	Pyrosequencing (PSQ HS96MA) Ultrasound of carotid arteries (Sonoscape S6)
Data format	Raw and graphs
Parameters for data collection	Blood samples and ultrasound images of carotid arteries from 190 subjects with subclinical atherosclerosis from Novosibirsk, Russia and 61 from Almaty, Kazakhstan were collected
Description of data collection	Mitochondrial heteroplasmy level m.13513G > A, m.3336T > C, m.12315G > A, m.5178C > A, m.14459G > A, m.14846G > A were determined, and their association with carotid intima-media thickness was analysed
Data source location	Almaty, Kazakhstan Novosibirsk, Russia
Data accessibility	Raw data are provided with this article

**Table:** 

**Value of the Data** • The presented data are useful since demonstrate the associations of mutations of mitochondrial genome and proatherosclerotic phenotype that can be shown in ethnically different populations, but may vary between populations. • The data on association of mutations of mtDNA and carotid atherosclerosis are beneficial for scientists who investigate mitochondrial genetics of atherosclerosis in epidemiological trials at the population level. • The data may contribute to the development of further research aimed to investigate the role of the damage of mitochondrial genome in the pathogenesis of atherosclerosis and provide novel genetic markers of predisposition to atherosclerosis and molecular targets for further development of technologies aimed to prevention of age-related degenerative pathologies. • Associations of variants of mitochondrial heteroplasmy and carotid atherosclerosis can either be similar for different populations, or can have significant differences; therefore, it makes sense to carry out the search of the similar data in replication studies and verification studies in other populations, including ethnically different ones.

### 1 Data description

Table 1 describes clinical and laboratory characteristics of subjects from Russian population divided by sex (age, body mass index, arterial blood pressure, lipids profile, mean cIMT) indicating the differences between men and women.

**Table 1:** Clinical and laboratory characteristic of subjects from Russian population.

	Women	Men	p
Age, years	57.8 (2.4)	58.9 (3.1)	0.071
BMI, kg/m^2^	29.1 (4.9)	26.4 (4.5)	0.004*
SBP, mm Hg	125 (12)	128 (10)	0.221
DBP, mm Hg	81 (10)	83 (7)	0.371
Total cholesterol, mg/dL	234 (46)	219 (38)	0.081
HDL, mg/dL	49.5 (12.7)	45.4 (8.5)	0.088
LDL, mg/dL	163.8 (41.1)	151.2 (34.1)	0.117
TG, mg/dL	113.4 (68.1)	101.1 (46.3)	0.345
Mean cIMT	0.788 (0.109)	0.760 (0.132)	0.207

*, statistically significant difference at p < 0.05.

Table 2 describes clinical and laboratory characteristics of subjects from Kazakh population divided by sex (age, body mass index, arterial blood pressure, lipids profile, mean cIMT) indicating the differences between men and women.

**Table 2:** Clinical and laboratory characteristic of subjects from Kazakh population.

	Women	Men	p
Age, years	57.1 (6.0)	56.9 (6.1)	0.893
BMI, kg/m^2^	27.1 (3.1)	26.6 (2.6)	0.553
SBP, mm Hg	120 (16)	127 (12)	0.068
DBP, mm Hg	77 (10)	83 (5)	0.015*
Total cholesterol, mg/dL	207 (54)	239 (37)	0.016*
HDL, mg/dL	45.0 (19.1)	50.8 (16.2)	0.244
LDL, mg/dL	142.9 (51.8)	170.3 (40.1)	0.036*
TG, mg/dL	94.0 (48.2)	87.5 (24.1)	0.560
Mean cIMT	0.744 (0.107)	0.795 (0.105)	0.080

*, statistically significant difference at p < 0.05.

Table 3 demonstrates levels of mitochondrial heteroplasmy of subjects from Russian population indicating the differences between men and women.

**Table 3:** Levels of mitochondrial heteroplasmy in subjects from Russian population.

	Women	Men	p
m.13513G > A,%	24.0 (12.3)	20.8 (10.9)	0.194
m.3336T > C,%	4.3 (11.2)	2.7 (2.2)	0.436
m.12315G > A,%	36.7 (24.8)	31.6 (19.5)	0.283
m.5178C > A,%	6.4 (15.3)	13.3 (25.3)	0.045*
m.14459G > A,%	3.4 (1.7)	4.7 (7.3)	0.045*
m.14846G > A,%	16.6 (15.7)	20.3 (25.8)	0.303

*, statistically significant difference at p < 0.05.

Table 4 demonstrates levels of mitochondrial heteroplasmy of subjects from Kazakh population indicating the differences between men and women.

**Table 4:** Levels of mitochondrial heteroplasmy in subjects from Kazakh population.

	Women	Men	*P*
m.13513G > A,%	12.9 (5.7)	10.6 (5.7)	0.133
m.3336T > C,%	3.8 (5.0)	4.2 (5.5)	0.853
m.12315G > A,%	9.5 (10.3)	14.1 (10.4)	0.234
m.5178C > A,%	21.2 (10.8)	24.8 (3.6)	0.168
m.14459G > A,%	10.7 (13.0)	7.9 (11.6)	0.401
m.14846G > A,%	22.1 (12.5)	18.0 (9.4)	0.191

Table 5 presents clinical and laboratory characteristics (age, body mass index, arterial blood pressure, lipids profile, mean cIMT) of total groups of Russian and Kazakh subjects indicating statistical significance of the differences between populations.

**Table 5:** Comparison of clinical characteristics of Russian and Kazakh population-derived samples.

	Russian, total	Kazakh, total	p
Age, years	57.9 (2.6)	57.1 (6.0)	0.097
BMI, kg/m^2^	28.7 (4.8)	26.9 (2.9)	0.008*
SBP, mm Hg	126 (12)	122 (15)	0.075
DBP, mm Hg	81 (9)	79 (9)	0.118
Total cholesterol, mg/dL	232 (44)	218 (50)	0.040*
HDL, mg/dL	48.9 (12.2)	47.1 (18.5)	0.389
LDL, mg/dL	161.8 (40.3)	152 (49.4)	0.152
TG, mg/dL	111.4 (65.2)	91.7 (41.0)	0.027*
Mean cIMT	0.783 (0.113)	0.762 (0.108)	0.219

*, statistically significant difference at p < 0.05.

Table 6 presents levels of mitochondrial heteroplasmy of total groups of Russian and Kazakh subjects indicating statistical significance of the differences between populations.

**Table 6:** Comparison of levels of mitochondrial heteroplasmy of Russian and Kazakh population-derived samples.

	Russian, total	Kazakh, total	p
m.13513G > A,%	23.5 (12.1)	12.1 (5.8)	<0.001*
m.3336T > C,%	4.0 (10.3)	3.9 (5.1)	0.943
m.12315G > A,%	35.9 (24.1)	10.8 (10.4)	<0.001*
m.5178C > A,%	7.5 (17.4)	22.4 (9.2)	<0.001*
m.14459G > A,%	3.6 (3.3)	9.7 (12.5)	<0.001*
m.14846G > A,%	17.2 (17.6)	20.6 (11.5)	0.175

*, statistically significant difference at p < 0.05.

The analysis of samples derived from Russian (n = 190) and Kazakh (n = 61) populations demonstrates that levels of variants of heteroplasmy m.13513G > A and m.12315G > A mtDNA were significantly higher in Russian group, and heteroplasmy level of m.5178C > A and m.14459G > A was significantly higher in Kazakh group.

The association of analysed variants of mitochondrial heteroplasmy with mean carotid IMT of subjects from Russian and Kazakh populations in total groups and in men and women separately is presented in Table 7.

**Table 7:** Correlation of mean carotid IMT and mitochondrial heteroplasmy level in Russian and Kazakh population-derived samples.

Variable	Russian			Kazakh		
	Total	Women	Men	Total	Women	Men
m.13513G > A	−0.235 0.001*	−0.313 <0.001*	−0.087 0.646	−0.412 0.001*	−0.445 0.005*	−0.276 0.214
m.3336T > C	0.030 0.686	0.018 0.819	0.232 0.217	−0.031 0.861	−0.177 0.397	0.208 0.564
m.12315G > A	−0.018 0.808	−0.021 0.797	−0.053 0.779	0.262 0.128	0.420 0.036*	−0.235 0.514
m.5178C > A	−0.078 0.286	0.084 0.291	−0.476 0.008*	−0.045 0.747	−0.106 0.537	−0.039 0.876
m.14459G > A	0.001 0.985	−0.073 0.361	0.111 0.558	0.011 0.932	0.033 0.844	0.047 0.837
m.14846G > A	−0.025 0.727	−0.016 0.839	−0.026 0.891	0.081 0.546	0.062 0.720	0.263 0.237

r, Pearson's correlation coefficient and significance of correlation are shown.

*, statistical significance at p < 0.05.

Pearson's correlation analysis revealed significant association of mitochondrial heteroplasmy m.13513G > A with mean carotid IMT in both Russian and Kazakh groups. Upon subdivision of groups by sex, this correlation was significant only in women in both population-derived samples. In addition, negative correlation of m.5178C > A mitochondrial heteroplasmy with cIMT was found in male samples from Russian population, and positive correlation of m.12315G > A mitochondrial heteroplasmy was found in females from Kazakhstan. We have found no correlations of variants of mitochondrial heteroplasmy with traditional cardiovascular risk factors such as age, body mass index, blood pressure, blood cholesterol, triglycerides, high-density and low-density lipoprotein cholesterol in both groups.

**Fig. 1:**
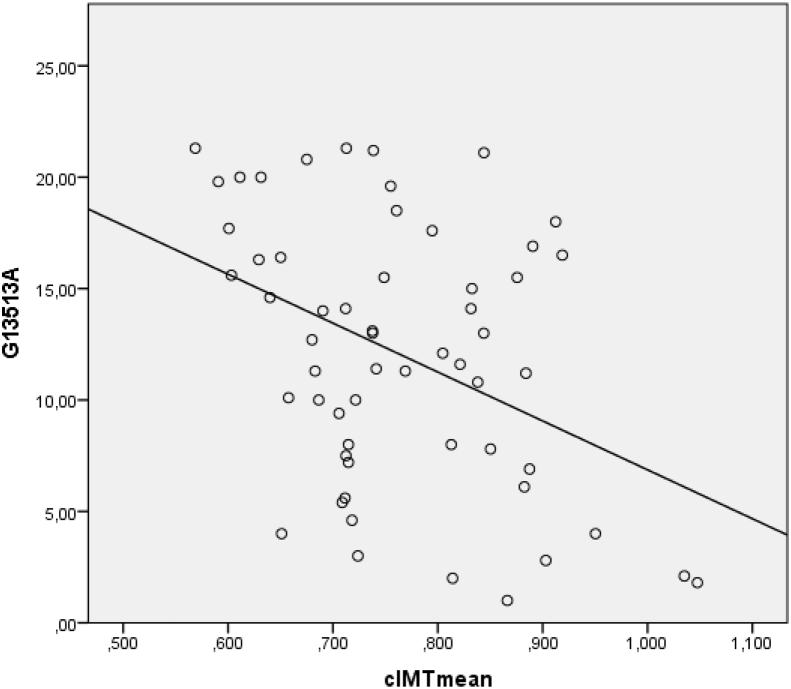
Correlation of mitochondrial heteroplasmy m.13513G > A and mean cIMT in Kazakh population-derived sample.

**Fig. 2:**
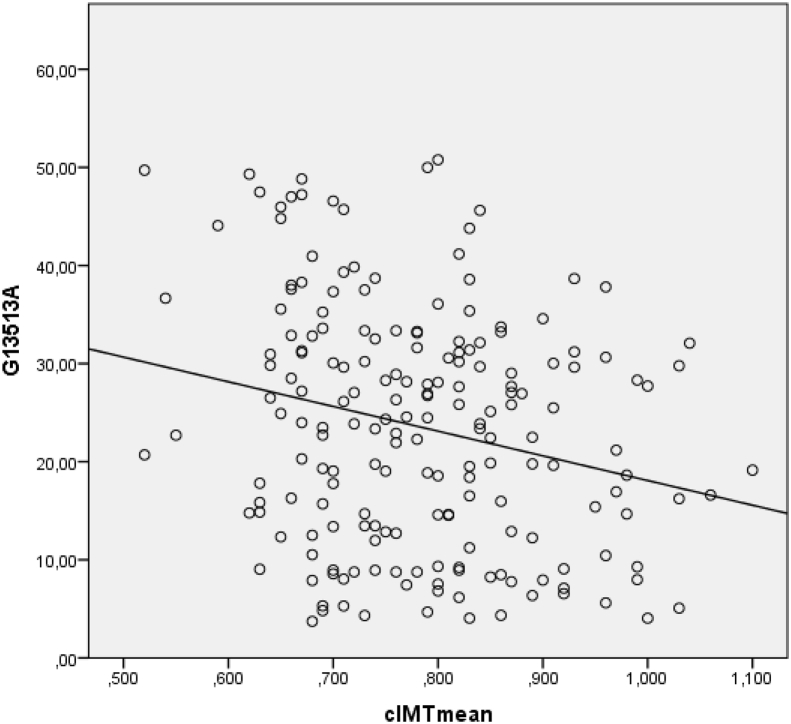
Correlation of mitochondrial heteroplasmy m.13513G > A and mean cIMT in Russian population-derived sample.

Graphs of correlation of mitochondrial heteroplasmy m.13513G > A and mean carotid IMT in Kazakh and Russian population-derived samples are presented on Fig. 1, Fig. 2, respectively.

Dataset is presented as supplementary material.

### 2 Experimental design, materials and methods

The association of mitochondrial genome variation with cardiovascular disease is an extremely relevant issue of modern scientists nowadays [[1], [2], [3], [4], [5]]. Our previous data demonstrate the significant differences of mitochondrial heteroplasmy between unaffected and atherosclerotic areas of human aortic intima, and detected heteroplasmic variants of mitochondrial genome statistically related to atherosclerosis [6,7]. Furthermore, we have shown the association of several heteroplasmic mtDNA variants with carotid atherosclerosis in samples derived from Moscow population [[8], [9], [10]]. Our recent data show that the mutations of the mitochondrial genome are differently related to cardiovascular disease in genetically and clinically diverse populations, Russian and Mexican ones [11]. In this way, it seems to be important to make comparisons between ethnically different populations that have similar socio-economic conditions.

In this article the association of heteroplasmy level of mitochondrial mtDNA variants with carotid atherosclerosis in Russian and Kazazh population-derived samples, was demonstrated. In total, data on 251 participants were analysed (190 participants from Novosibirsk, Russia, and 61 participants from Almaty, Kazakhstan). Men and women were aged 50–70 years old, and females were included on additional criterion, namely, more than 5 years after spontaneous (non-surgical) menopause. All participants were free of clinical manifestations of atherosclerosis-related diseases. The protocol met the standards of Declaration of Helsinki (the revised version of 1975 and the amendments of 1983, 1989, and 1996), and has been approved by the local ethical committee at the Institute for Atherosclerosis Research; all participants have provided written informed consent prior to inclusion.

Carotid arteries were examined by high-resolution B-mode ultrasound using a SonoScape S6 scanner (SonoScape, China) equipped with a 7.5 MHz linear array probe. Both left and right common carotid arteries were visualized in different projections (anterolateral, lateral, and posterolateral). The cIMT measurements were performed on the first centimeter of common carotid arteries before carotid bulb using dedicated M'Ath PACS software (IMT, France). The mean value of these measures was considered as an integral measure of intima-media thickness (mean cIMT).

Phenol-chloroform extraction was used for mitochondrial DNA isolation from blood leukocytes [12]. Polymerase chain reaction (PCR) was used to obtain DNA fragments covering the investigated variant nucleotide [6]. Pyrosequencing of PCR fragments was carried out by device PSQ HS96MA (Biotage, Sweden) to determine the heteroplasmy level as a percent of mtDNA mutant copies, as described elsewhere [6,13].

Data processing was performed by the IBM SPSS Statistics software, version 20.0 (SPSS IBM Inc., USA). Data are expressed in terms of means and standard deviation. The significance of differences was defined at the 95% level of confidence. Pearson's correlation analysis was used to evaluate the association of mitochondrial heteroplasmy with cIMT.
